# Dementia and Alzheimer's Risk Factors: Understanding the Role of Individual Values

**DOI:** 10.1002/alz70858_101443

**Published:** 2025-12-25

**Authors:** Akum Dhillon

**Affiliations:** ^1^ Burjeel Holdings, Abu Dhabi, Abu Dhabi, United Arab Emirates

## Abstract

**Background:**

Alzheimer's disease (AD) accounts for 60‐70% of dementia cases. Unlike other types, AD is characterized by amyloid plaques and neurofibrillary tangles causing neurodegeneration (Kalaria, 2018; McKeith et al., 2005). The predictable course begins with memory loss and progresses to severe cognitive impairment. With significant social and economic impact, addressing AD's burden in regions like MENA is crucial for effective public health planning and targeted interventions.

**Methods:**

A comprehensive literature search was conducted using reputable databases, including PubMed and Scopus. The search parameters were refined with specific keywords and Boolean operators to ensure relevance. A multi‐step screening process reviewed titles, abstracts, and full texts to collect relevant information on methodology, sample size, interventions, outcomes, and key findings.

**Results:**

Our literature review found that dementia prevalence, including AD, has risen 3% since 1990 to 777.6 per 100,000 people in MENA by 2019. By 2050, 152 million people will live with dementia globally. MENA shows regional variations: Turkey and Bahrain have higher rates than the UAE and Afghanistan. Dementia costs $10.4‐13.9 billion annually in MENA, including healthcare expenses and informal care. Healthcare disparities hinder AD diagnosis and treatment in low‐income countries like Yemen and Afghanistan due to limited access to diagnostic tools, trained providers, and cultural stigma. In contrast, GCC nations have advanced early diagnosis and care systems. This highlights the need for policies that expand dementia care infrastructure, improve accessibility to diagnostic tools, and train healthcare providers to address AD management demands in under‐resourced areas.

**Conclusion:**

The MENA region requires urgent attention to understand dementia and AD causes. It is crucial to investigate public and healthcare professionals' understanding and attitudes towards AD, as well as proposed solutions to address gaps in the region. This study aims to fill this knowledge gap.

**References**:

1. Kalaria, R. N. (2018). The pathology and pathophysiology of vascular dementia. *Neuropharmacology*, 134, 226‐239.

2. McKeith, I. G., et al. (2005). Diagnosis and management of dementia with Lewy bodies: Third report of the DLB Consortium. *Neurology*, 65(12), 1863‐1872.

